# A New Stochastic Split-Step *θ*-Nonstandard Finite Difference Method for the Developed SVIR Epidemic Model with Temporary Immunities and General Incidence Rates

**DOI:** 10.3390/vaccines10101682

**Published:** 2022-10-09

**Authors:** Abdulwasea Alkhazzan, Jungang Wang, Yufeng Nie, Khalid Hattaf

**Affiliations:** 1 School of Mathematics and Statistic, Northwestern Polytechnical University, Xi’an 710072, China; 2 Department of Mathematics, College of Science, Sana’a University, Sana’a P.O. Box 1247, Yemen; 3 Equipe de Recherche en Modélisation et Enseignement des Mathématiques (ERMEM), Centre Régional des Métiers de l’Education et de la Formation (CRMEF), Derb Ghalef, Casablanca 20340, Morocco; 4 Laboratory of Analysis, Modeling and Simulation (LAMS), Faculty of Sciences Ben M’Sick, Hassan II University of Casablanca, P.O. Box 7955 Sidi Othman, Casablanca 20000, Morocco

**Keywords:** stochastic SVIR epidemic model, extinction, persistence, SSTM method, SSSTNSFD method, temporary immunity, general incidence rate

## Abstract

In this paper, an SVIR epidemic model with temporary immunities and general incidence rates is constructed and analyzed. By utilizing Lyapunov functions, we prove the existence and uniqueness of the positive global solution of the constructed model, as well as the sufficient conditions of extinction and persistence of disease, are provided. Due to the difficulty of obtaining the analytical solution to our model, we construct two numerical schemes to generate an approximate solution to the model. The first one is called the split-step θ-Milstein (SSTM) method, and the second one is called the stochastic split-step θ-nonstandard finite difference (SSSNSFD) method, which is designed by merging split-step θ method with stochastic nonstandard finite difference method for the first time in this paper. Further, we prove the positivity, boundedness, and stability of the SSSTNSFD method. By employing the two mentioned methods, we support the validity of the studied theoretical results, as well, the effect of the length of immunity periods, parameters values of the incidence rates, and noise on the dynamics of the model are discussed and simulated. The increase in the size of time step size plays a vital role in revealing the method that preserves positivity, boundedness, and stability. To this end, a comparison between the proposed numerical methods is carried out graphically.

## 1. Introduction

Stochastic modeling is considered as one of the widely used strategies in the modeling of infectious diseases for the purpose of studying the dynamics of the diseases. Moreover, it is observed that stochastic models are usually more informative than deterministic models, where a deterministic model can predicts only a single result based on a given set of circumstances. In contrast, a stochastic model predicts a set of possible outcomes. In recent years, many researchers have proposed many mathematical models by using stochastic differential equations to describe the dynamics of epidemics (see, e.g., Refs. [1,2,3,4]). However, to obtain more realistic systems of population interactions, the authors included the temporal delays in such models and investigated their dynamics properties (see, e.g., Refs. [5,6,7]).

The vaccination can play an important role in controlling the diseases because it lowers the reproduction number and possibly decreases the number of infected individuals in the endemic area. As is known and confirmed that some vaccines confer a lifetime immunity against infection, while others provide only temporary immunity. So, Infection-induced or vaccination-induced immunity period is considered as one of the delay factors used in constructing the epidemic models, which were used by authors in many published papers (see, e.g., Refs. [8,9,10]). Due to the effective strategy of vaccines for controlling diseases, the authors in [11] established the stochastic SVIR epidemic model based on the corresponding deterministic model, which was constructed and analyzed in [12]. In particular, the authors in [11] proved the existence and uniqueness of the positive global solution of the following SVIR epidemic model:

(1) $$\left\{\begin{array}{cc} dS(t) & =\left[\mu -\left(\mu +\alpha \right)S\left(t\right)-\beta_{1}SI\right]dt+\sigma_{1}SdW_{1},\\ dV(t) & =\left[\alpha S\left(t\right)-\left(\gamma_{2}+\mu \right)V\left(t\right)-\beta_{2}VI\right]dt+\sigma_{2}VdW_{2},\\ dI(t) & =\left[\beta_{1}SI+\beta_{2}VI-\left(\gamma_{1}+\mu \right)I\left(t\right)\right]dt+\sigma_{3}IdW_{3},\\ dR(t) & =\left[\gamma_{1}I\left(t\right)+\gamma_{2}V\left(t\right)-\mu R\left(t\right)\right]dt+\sigma_{4}RdW_{4}. \end{array}\right.$$

Moreover, they provided sufficient conditions for the extinction and persistence of the disease. The studied stochastic model (1) was constructed with bilinear incidence rates and without considering the temporary immunity.

In epidemic modeling, it is well known that incidence rates are crucial in ensuring that epidemic models provide a reasonable explanation of infectious disease dynamics. Many scholars suggest using the nonlinear incidence rate instead of standard incidence rates and bilinear incidence rates in the transmission process of disease in order to analyze the mechanism of disease transmission better and provide a more flexible model for dealing with realistic data [13]. Recently, Hattaf et al. [14] introduced a general functional response $F\left(S,I\right)=\frac{\beta S}{1+\lambda_{1}S+\lambda_{2}I+\lambda_{3}SI},$ where $\lambda_{1}$,$\lambda_{2}$,$\lambda_{3}$≥0 are the saturation factors measuring the psychological or inhibitory effect. This function enables us to derive several types of incidence rates existing in the literature. For example, the bilinear incidence rate F(S,I)=βS is obtained if $\lambda_{1}=\lambda_{2}=\lambda_{3}=0,$ (see [15]). The saturated incidence function $F\left(S,I\right)=\frac{\beta S}{1+\lambda_{1}S}$ is obtained if $\lambda_{2}=\lambda_{3}=0,$ or $F\left(S,I\right)=\frac{\beta S}{1+\lambda_{2}I}$ is obtained if $\lambda_{1}=\lambda_{3}=0,$ (see [16,17]). Beddington-DeAngelis functional response $F\left(S,I\right)=\frac{\beta S}{1+\lambda_{1}S+\lambda_{2}I}$ is obtained if $\lambda_{3}=0,$ (see [18]). Crowley-Martin functional response $F\left(S,I\right)=\frac{\beta S}{1+\lambda_{1}S+\lambda_{2}I+\lambda_{1}\lambda_{2}SI}$ is obtained if $\lambda_{3}=\lambda_{1}\lambda_{2},$ (see [19]).

On the other hand, the duration of immunity is one of the most critical aspects of disease and vaccine that effectively affects the impact of vaccines on public health for population communities. It was observed that the duration of immunity acquired by the individual against infectious diseases ranges from almost non-existent to lifelong [20]. For instance, both of vaccine of varicella [21] and the vaccine of pertussis [22] provide only temporary immunity for the vaccinated individual against the infectious. For many infectious diseases, the immunity (whether infection-induced immunity or vaccination-induced immunity) wanes either due to the loss of immune memory or evolution of the disease itself [23].

Motivated by the facts mentioned above, we develop in this article the model (1) by modeling the disease incidence rates via general functional responses and incorporating the temporary immunities. Regarding temporary immunities, we assume that, due to loss of immune memory, the evolution of the disease itself, or any other reasons, a fraction of recovered infected individuals may lose their infection-induced immunity and returns to the susceptible compartment. Also, a fraction of recovered vaccinated individuals may lose their vaccination-induced immunity and then moves to the vaccinees compartment to have booster or additional doses for enhancing or restoring the protection, which faded over time after the primary series vaccination was taken.

To distinguish between the booster and additional doses, the authors in [24] mentioned that Booster doses are given for those individuals who responded adequately to primary series vaccination in order to restore protection after it would have waned, while additional doses are given for those immunocompromised individuals who did not respond adequately to the primary series vaccination.

Specifically, the developed stochastic SVIR model is described by the following stochastic it$\hat{o}$ equations:

(2) $$\left\{\begin{array}{cc} dS(t) & =[\mu -\left(\mu +\alpha \right)S\left(t\right)-F_{1}\left(S\left(t\right),I\left(t\right)\right)+\gamma_{1}e^{-\mu \tau_{1}}I\left(t-\tau_{1}\right)]dt+\sigma_{1}S\left(t\right)dW_{1}\left(t\right),\\ dV(t) & =\left[\alpha S\left(t\right)-\left(\gamma_{2}+\mu \right)V\left(t\right)-F_{2}\left(V\left(t\right),I\left(t\right)\right)+\gamma_{2}e^{-\mu \tau_{2}}V\left(t-\tau_{2}\right)\right]dt+\sigma_{2}V\left(t\right)dW_{2}\left(t\right),\\ dI(t) & =\left[F_{1}\left(S\left(t\right),I\left(t\right)\right)+F_{2}\left(V\left(t\right),I\left(t\right)\right)-\left(\gamma_{1}+\mu \right)I\left(t\right)\right]dt+\sigma_{3}I\left(t\right)dW_{3}\left(t\right),\\ dR(t) & =\left[\gamma_{1}I\left(t\right)+\gamma_{2}V\left(t\right)-\gamma_{1}e^{-\mu \tau_{1}}I\left(t-\tau_{1}\right)-\gamma_{2}e^{-\mu \tau_{2}}V\left(t-\tau_{2}\right)-\mu R\left(t\right)\right]dt+\sigma_{4}RdW_{4}\left(t\right), \end{array}\right.$$

where the letters S,I and R represent the densities of susceptible, infected, and recovered individuals, respectively, whilst the letter V represents the density of the individuals who have begun the vaccination process. The total size of population will be represented by N (i.e., N=S+V+I+R). The Biological meanings of all parameters in model (2) are listed in Table 1. In our model, we have two terms to denote the temporary immunities. The first term $e^{-\mu \tau_{1}}I\left(t-\tau_{1}\right)$ indicates those individuals who survive natural death after they have become infected and then become susceptible due to the loss of infection-induced immunity for a specific time $\tau_{1}>0$. The second term $e^{-\mu \tau_{2}}V\left(t-\tau_{2}\right)$ indicates to those individuals who survive from natural death after they have completed their primary vaccine series and then move to the vaccinees compartment (V) to have booster or additional doses due to the loss of vaccination-induced immunity for a specific time $\tau_{2}>0.$ However, due to the possibility that the vaccinees individuals have some partial immunity during the vaccination process, it is assumed that $\beta_{2}$ less than $\beta_{1}$.

For $\lambda_{i}\geq 0,\eta_{i}\geq 0,\forall i\in N_{1}^{3},$ the specific nonlinear functions $F_{1},F_{2}$ represent the incidence rates, in which they have the following forms:

$$\begin{array}{cc} & F_{1}\left(S\left(t\right),I\left(t\right)\right)=\frac{\beta_{1}SI}{1+\lambda_{1}S+\lambda_{2}I+\lambda_{3}SI},\;\;F_{2}\left(V\left(t\right),I\left(t\right)\right)=\frac{\beta_{2}VI}{1+\eta_{1}V+\eta_{2}I+\eta_{3}VI}, \end{array}$$

and satisfying the below properties:

- $F_{i}:R_{+}^{2}\to R_{+}$ are differentiable functions, where $F_{i}\left(0,I\right)=F_{1}\left(S,0\right)=F_{1}\left(V,0\right)=0,\forall \;\;S,V,I\geq 0,$ s.t. i∈{1,2};
- $\lim_{I\to 0^{+}}{\bar{F}}_{1}\left(S,I\right),\lim_{I\to 0^{+}}{\bar{F}}_{2}\left(S,I\right)$ are exist and positive ∀S,V>0, where ${\bar{F}}_{1}\left(S,I\right)=\frac{F_{1}\left(S,I\right)}{I}$ and ${\bar{F}}_{2}\left(V,I\right)=\frac{F_{1}\left(V,I\right)}{I};$
- $\frac{\partial F_{1}}{\partial S}\left(S,I\right)>0$ and $\frac{\partial {\bar{F}}_{1}}{\partial I}\left(S,I\right)\leq 0,\forall S,I\geq 0;$
- $\frac{\partial F_{2}}{\partial V}\left(V,I\right)>0$ and $\frac{\partial {\bar{F}}_{2}}{\partial I}\left(V,I\right)\leq 0,\forall \;V,I\geq 0;$
- $F_{1}\left(S,I\right)\leq \beta_{1}SI$ and $F_{2}\left(V,I\right)\leq \beta_{2}VI,\forall \;S,V,I\geq 0.$

In addition, for $i\in N_{1}^{4},W_{i}$ are independent standard Brownian motions defined on a complete probability space with a filtration $\left(\Omega ,f,P,{\left\{f_{t}\right\}}_{t\geq 0}\right)$ satisfying the usual conditions (i.e., it is right continuous and $f_{0}$ contains all P−null sets), and $\sigma_{i}$ are their intensities.

In looking at the structure of the model (2), we observe that the term R is absent in the first three equations. Therefore, the fourth equation can be ignored from the model without loss of generality. Thus, we here only debate the following model:

(3) $$\left\{\begin{array}{cc} dS(t) & =[\mu -\left(\mu +\alpha \right)S\left(t\right)-F_{1}\left(S\left(t\right),I\left(t\right)\right)+\gamma_{1}e^{-\mu \tau_{1}}I\left(t-\tau_{1}\right)]dt+\sigma_{1}S\left(t\right)dW_{1}\left(t\right),\\ dV(t) & =\left[\alpha S\left(t\right)-\left(\gamma_{2}+\mu \right)V\left(t\right)-F_{2}\left(V\left(t\right),I\left(t\right)\right)+\gamma_{2}e^{-\mu \tau_{2}}V\left(t-\tau_{2}\right)\right]dt+\sigma_{2}V\left(t\right)dW_{2}\left(t\right),\\ dI(t) & =\left[F_{1}\left(S\left(t\right),I\left(t\right)\right)+F_{2}\left(V\left(t\right),I\left(t\right)\right)-\left(\gamma_{1}+\mu \right)I\left(t\right)\right]dt+\sigma_{3}I\left(t\right)dW_{3}\left(t\right), \end{array}\right.$$

where R=N−S−V−I. Obviously, the disease free equilibrium of model (3) is $E_{0}=\left(S_{0},V_{0},I_{0}\right)=\left(\frac{\mu }{\alpha +\mu },\frac{\alpha \mu }{c_{2}},0\right),$ where $c_{2}=\left(\gamma_{2}\left(1-e^{-\mu \tau_{2}}\right)+\mu \right)\left(\alpha +\mu \right).$

After formulating the developed SVIR model (3), we reveal about the contributions of the current study, which are listed as follows:

- Investigating the existence and uniqueness of the positive global solution of the model (3);
- Establishing the sufficient conditions for the extinction and persistence of disease;
- Designing a novel numerical method to approximate the solution of our model and comparing its performance with another method. This new method can be used to approximate the solution of other stochastic delayed epidemic models;
- Discussing the effect of the length of immunity periods, parameter values of the incidence rates and noise on the dynamics of the model.

The strategy of this paper is broken down as follows: Section 2 is devoted to the stochastic analysis of the model (3) and divided into three sections: in Section 2.1, the existence and uniqueness of the positive global solution of the model (3) are investigated (see Theorem 1). According to Section 2.2, the stochastic reproduction number is defined and used to provide sufficient conditions for the extinction of disease (see Theorem 2). At the same time, sufficient conditions for the persistence of disease are provided in Section 2.3 (see Theorem 3). Section 3 is allocated to the numerical analysis of the developed model and involves three sections: In Section 3.1, we construct SSTM scheme for the model (3). In Section 3.2, we design and analyze a new stochastic method, namely, SSSTNSFD scheme for (3) is constructed and analyzed. With regard to the validity and effectiveness of the obtained results, both of the two constructed methods are employed to support those results graphically in Section 3.3. Finally, the conclusion of the study is discussed in Section 4.

## 2. Stochastic Model Analysis

This section is dedicated to show that model (3) has a positive global solution. Furthermore, by establishing a stochastic reproduction number ($R_{0}^{s}$), we provide sufficient conditions for extinction and persistence of the disease. For some upcoming proofs, we need to reformulate the two functions ${\bar{F}}_{1}$ and ${\bar{F}}_{2}$ as follows:

(4) $$\begin{array}{cc} {\bar{F}}_{1} & =\frac{\beta_{1}S}{1+\lambda_{1}S+\lambda_{2}I+\lambda_{3}SI}=\frac{\beta_{1}\mu }{(1+\lambda_{1})\mu +\alpha }-\frac{\beta_{1}\left(\alpha +\mu \right)}{\left(1+\lambda_{1}S+\lambda_{2}I+\lambda_{3}SI\right)\left(\left(1+\lambda_{1}\right)\mu +\alpha \right)}\left(\frac{\mu }{\alpha +\mu }-S\right)\\ & -\frac{\beta_{1}\lambda_{2}\mu }{\left(1+\lambda_{1}S+\lambda_{2}I+\lambda_{3}SI\right)\left(\left(1+\lambda_{1}\right)\mu +\alpha \right)}I-\frac{\beta_{1}\lambda_{3}\mu }{\left(1+\lambda_{1}S+\lambda_{2}I+\lambda_{3}SI\right)\left(\left(1+\lambda_{1}\right)\mu +\alpha \right)}SI, \end{array}$$

(5) $$\begin{array}{cc} {\bar{F}}_{2} & =\frac{\beta_{2}V}{1+\eta_{1}V+\eta_{2}I+\eta_{3}VI}=\frac{\beta_{2}\alpha \mu }{\eta_{1}\alpha \mu +c_{2}}-\frac{\beta_{2}c_{2}}{\left(1+\eta_{1}V+\eta_{2}I+\eta_{3}VI\right)\left(\eta_{1}\alpha \mu +c_{2}\right)}\left(\frac{\alpha \mu }{c_{2}}-V\right)\\ & -\frac{\beta_{2}\eta_{2}\alpha \mu }{\left(1+\eta_{1}V+\eta_{2}I+\eta_{3}VI\right)\left(\eta_{1}\alpha \mu +c_{2}\right)}I-\frac{\beta_{2}\eta_{3}\alpha \mu }{\left(1+\eta_{1}V+\eta_{2}I+\eta_{3}VI\right)\left(\eta_{1}\alpha \mu +c_{2}\right)}VI. \end{array}$$

Now, from (4), we deduce

(6) $$\begin{array}{cc} {\bar{F}}_{1} & \leq \frac{\beta_{1}\mu }{(1+\lambda_{1})\mu +\alpha }, \end{array}$$

(7) $$\begin{array}{cc} {\bar{F}}_{1} & \geq \frac{\beta_{1}\mu }{(1+\lambda_{1})\mu +\alpha }-\frac{\beta_{1}\mu }{(1+\lambda_{1})\mu +\alpha }\left(\lambda_{2}+\lambda_{3}\frac{\mu }{\alpha +\mu }\right)I. \end{array}$$

Also, from (5), we conclude

(8) $$\begin{array}{cc} {\bar{F}}_{2} & \leq \frac{\beta_{2}\alpha \mu }{\eta_{1}\alpha \mu +c_{2}}, \end{array}$$

(9) $$\begin{array}{cc} {\bar{F}}_{2} & \geq \frac{\beta_{2}c_{2}V}{\eta_{1}\alpha \mu +c_{2}}-\frac{\beta_{2}\alpha \mu }{\eta_{1}\alpha \mu +c_{2}}\left(\eta_{2}+\eta_{3}\frac{\alpha \mu }{c_{2}}\right)I. \end{array}$$

### 2.1. Existence and Uniqueness of Positive Global Solution

Let $\tau =\max\{\tau_{1},\tau_{2}\},$ and we define $R_{+}^{3}=\left\{\left(x_{1},x_{2},x_{3}\right)\in R^{3}:x_{i}>0,i\in N_{1}^{3}\right\}$ and $Q=C(\left[-\tau ,0\right],R_{+}^{3})$ be the Banach space of continuous functions mapping from the interval [−τ,0] into $R_{+}^{3}$ and is equipped by the norm $\left||\Psi |\right|=\sup_{-\tau \leq \xi \leq 0}\left|\Psi \left(\xi \right)\right|.$ Biologically, we assume the initial conditions of model (3) to be:

(10) $$\left\{\begin{array}{c} S\left(\xi \right)=\Psi_{1}\left(\xi \right),V\left(\xi \right)=\Psi_{2}\left(\xi \right),I\left(\xi \right)=\Psi_{3}\left(\xi \right),\\ \Psi_{i}\left(\xi \right)>0,\;\;\xi \in \left[-\tau ,0\right],\\ (\Psi_{1}.\Psi_{2},\Psi_{3})\in Q. \end{array}\right.$$

According to the following theorem, we can deduce that there is a unique positive global solution of model (3) with the described conditions (10).

**Theorem** **1.** *For any initial value $\left(S\left(0\right),V\left(0\right),I\left(0\right)\right)\in R_{+}^{3},$ model (3) admits a unique positive global solution given by (S(t),V(t),I(t)),∀t≥0, and with probability one, the solution will remain in $R_{+}^{3}$ almost surely (a.s.).*

**Proof.**  It is clear that the coefficients of the model (3) are locally Lipschits continuous. Based on that, we can say that for any initial value $\left(S\left(0\right),V\left(0\right),I\left(0\right)\right)\in R_{+}^{3},$ a unique local positive solution (S(t),V(t),I(t)) is exist on $t\in [-\tau ,\tau_{e}),$ where $\tau_{e}$ represents the explosion time. In this position, we only show that $\tau_{e}=\infty$ a.s. to prove that the solution (S(t),V(t),I(t)) is nonegative global solution of model (3). For this end, we need to define the following stopping time

$$\tau^{*}=\inf\left\{t\in \left[-\tau ,\tau_{e}\right):S\left(t\right)\leq 0,V\left(t\right)\leq 0\;\;\mathrm{or}\;\;I\left(t\right)\leq 0\right\},$$

where infϕ=∞ (ϕ is defined as empty set). Obviously, we observe that $\tau^{*}\leq \tau_{e},$ so if it is proven that $\tau^{*}=\infty ,$ then this implies $\tau_{e}=\infty$ which in turn means that the solution $\left(S\left(t\right),V\left(t\right),I\left(t\right)\right)\in R_{+}^{3},\forall t\geq 0$ a.s. Let us assume that $\tau^{*}<\infty ,$ then there exists a constant T>0, s.t. $P(\tau^{*}<T)>0.$ Now, we define a fundamental $C^{2}-$function $G_{1}:R_{+}^{3}\to R_{+},$ via the below formulation:

(11) $$\begin{array}{ccc} G_{1}\left(S\left(t\right),V\left(t\right),I\left(t\right)\right) & = & \ln S+\ln V+\ln I. \end{array}$$

To complete the proof, we apply It$\hat{o}$’s formula on $G_{1},\forall \;\;t\in \left[0,\tau^{*}\right]$ and all $\omega \in \{\tau^{*}<T\}$ as follows:

(12) $$\begin{array}{ccc} dG_{1} & = & [\frac{\mu }{S}-\left(\mu +\alpha \right)-\frac{F_{1}}{S}+\frac{\gamma_{1}e^{-\mu \tau_{1}}I\left(t-\tau_{1}\right)}{S}+\frac{\alpha S}{V}-\left(\gamma_{2}+\mu \right)-\frac{F_{2}}{V}+\frac{\gamma_{2}e^{-\mu \tau_{2}}V\left(t-\tau_{2}\right)}{V}\\ & + & {\bar{F}}_{1}+{\bar{F}}_{2}-\left(\gamma_{1}+\mu \right)-\frac{\sigma_{1}^{2}+\sigma_{2}^{2}+\sigma_{3}^{2}}{2}]dt+\sigma_{1}dW_{1}+\sigma_{2}dW_{2}+\sigma_{3}dW_{3}\\ & \geq & -\left[3\mu +\alpha +\gamma_{1}+\gamma_{2}+\frac{\sigma_{1}^{2}+\sigma_{2}^{2}+\sigma_{3}^{2}}{2}+\left(\beta_{1}+\beta_{2}\right)I\right]dt+\sigma_{1}dW_{1}+\sigma_{2}dW_{2}+\sigma_{3}dW_{3}. \end{array}$$

Integrating both sides of (12) over [0,t], we get

(13) $$\begin{array}{ccc} G_{1}\left(t\right)-G_{1}\left(0\right) & \geq & -(3\mu +\alpha +\gamma_{1}+\gamma_{2}+\frac{\sigma_{1}^{2}+\sigma_{2}^{2}+\sigma_{3}^{2}}{2})t\\ & - & \left(\beta_{1}+\beta_{2}\right)\int_{0}^{t}I\left(s\right)ds+\sigma_{1}W_{1}\left(t\right)+\sigma_{2}W_{2}\left(t\right)+\sigma_{3}W_{3}\left(t\right). \end{array}$$

According to the definition of $\tau^{*}$, we deduce that the solution of model (3) is always nonnegative on $\left[0,\tau^{*}\right)$ for all $\omega \in \{\tau^{*}<T\}$ as well as $S\left(\tau^{*}\right)=V\left(\tau^{*}\right)=I\left(\tau^{*}\right)=0.$ Thence, $\lim_{t\to \tau^{*}}G_{1}\left(t\right)=-\infty .$ By letting *t* tends to $\tau^{*}$ in both sides of (13), we obtain

(14) $$\begin{array}{ccc} -\infty \geq & - & \left(3\mu +\alpha +\gamma_{1}+\gamma_{2}+\frac{\sigma_{1}^{2}+\sigma_{2}^{2}+\sigma_{3}^{2}}{2}\right)\tau^{*}+\left(\beta_{1}+\beta_{2}\right)\int_{0}^{\tau^{*}}I\left(s\right)ds\\ & + & \sigma_{1}W_{1}\left(\tau^{*}\right)+\sigma_{2}W_{2}\left(\tau^{*}\right)+\sigma_{3}W_{3}\left(\tau^{*}\right)>-\infty , \end{array}$$

and this produces a contradiction. Consequently, $\tau^{*}=\tau_{e}=+\infty \;\;a.s,$ which means that the solution (S(t),V(t),I(t)) of model (3) is positive global solution a.s. This completes the proof. □

### 2.2. Extinction of Disease

**Lemma** **1.** *Let (S(t),V(t),I(t)) be the solution of model (3) with initial value $S\left(0\right)>0,V\left(\zeta_{2}\right)\geq 0$ and $I(\zeta_{1})\geq 0$, $\forall \zeta_{1}\in \left[-\tau_{1},0\right)$ and $\zeta_{2}\in \left[-\tau_{2},0\right)$ with V(0)>0 and I(0)>0, then*

$$\begin{array}{c} \lim_{t\to \infty }\frac{S\left(t\right)+V\left(t\right)+I\left(t\right)+\gamma_{1}e^{-\mu t}\int_{t-\tau_{1}}^{t}e^{\mu s}I\left(s\right)ds+\gamma_{2}e^{-\mu t}\int_{t-\tau_{2}}^{t}e^{\mu s}V\left(s\right)ds}{t}=0\;\;a.s. \end{array}$$

*Moreover,*

$$\lim_{t\to \infty }\frac{S(t)}{t}=\lim_{t\to \infty }\frac{V(t)}{t}=\lim_{t\to \infty }\frac{I(t)}{t}=0,$$

*and*

$$\lim_{t\to \infty }\frac{e^{-\mu t}\int_{t-\tau_{1}}^{t}e^{\mu s}I\left(s\right)ds}{t}=\lim_{t\to \infty }\frac{e^{-\mu t}\int_{t-\tau_{2}}^{t}e^{\mu s}V\left(s\right)ds}{t}=0\;\;a.s.$$

**Lemma** **2.** *Let (S(t),V(t),I(t)) be the solution of model (3) with any given initial value $S\left(0\right)>0,V\left(\zeta_{2}\right)\geq 0$ and $I(\zeta_{1})\geq 0$, $\forall \zeta_{1}\in \left[-\tau_{1},0\right)$ and $\zeta_{2}\in \left[-\tau_{2},0\right)$ with V(0)>0 and I(0)>0, then*

$$\lim_{t\to \infty }\frac{\int_{0}^{t}S\left(s\right)dW_{1}}{t}=\lim_{t\to \infty }\frac{\int_{0}^{t}V\left(s\right)dW_{2}}{t}=\lim_{t\to \infty }\frac{\int_{0}^{t}I\left(s\right)dW_{3}}{t}=0\;\;a.s.$$

The proofs of Lemmas 1 and 2 are almost the same to those in Lemma 3.1 [10] and Lemma 2 [25]. Thus, herein, they can be omitted. Now, we define the reproduction number for model (3) as:

$$R_{0}^{s}=\frac{\beta_{1}\mu }{\left(\alpha +\left(1+\lambda_{1}\right)\mu \right)\left(\gamma_{1}+\mu +\frac{\sigma_{3}^{2}}{2}\right)}+\frac{\beta_{2}\alpha \mu }{\left(\eta_{1}\alpha \mu +c_{1}\right)\left(\gamma_{1}+\mu +\frac{\sigma_{3}^{2}}{2}\right)},$$

which is arguably the most significant quantity in infectious disease epidemiology that used for estimating the average number of new infections caused by an infectious individual in a population where a fraction of the susceptible individuals is vaccinated. Nonetheless, when noise does not exist, we get the reproduction number of the corresponding deterministic model as follows:

$$R_{0}^{d}=\frac{\beta_{1}\mu }{\left(\alpha +\left(1+\lambda_{1}\right)\mu \right)\left(\gamma_{1}+\mu \right)}+\frac{\beta_{2}\alpha \mu }{\left(\eta_{1}\alpha \mu +c_{1}\right)\left(\gamma_{1}+\mu \right)}.$$

For simplicity, the below notation is introduced:

$$\langle g\left(t\right)\rangle =\frac{1}{t}\int_{0}^{t}g\left(s\right)ds,\;\mathrm{for}\;\mathrm{any}\;\mathrm{integrable}\;\mathrm{function}\;g\;\mathrm{on}\;\;\left[0,\infty \right).$$

By utilizing the previous lemmas, sufficient conditions of extinction of disease can be obtained through the theorem below, which is one of the main results of the current article.

**Theorem** **2.** *Let (S(t),V(t),I(t)) be the solution of model (3) with initial value $S\left(0\right)>0,V\left(\zeta_{2}\right)\geq 0$ and $I(\zeta_{1})\geq 0$, $\forall \zeta_{1}\in \left[-\tau_{1},0\right)$ and $\zeta_{2}\in \left[-\tau_{2},0\right)$ with V(0)>0 and I(0)>0. If $R_{0}^{s}<1,$ then*

$$\begin{array}{c} \lim_{t\to \infty }\sup\frac{\ln I(t)}{t}\leq \left(\mu +\gamma_{1}+\frac{\sigma_{3}^{2}}{2}\right)\left(R_{0}^{s}-1\right)<0\;\;a.s. \end{array}$$

*Moreover,*

$$\lim_{t\to \infty }\langle S\left(t\right)\rangle \leq \frac{\mu }{\alpha +\mu },\;\;\;\;\;\;\;\;\lim_{t\to \infty }\langle V\left(t\right)\rangle \leq \frac{\alpha \mu }{c_{2}}.$$

**Proof.**  First, we define the function $G_{2}\left(S\left(t\right),I\left(t\right)\right)=S+I+\gamma_{1}e^{-\mu \tau_{1}}\int_{t-\tau_{1}}^{t}I\left(s\right)ds.$ Then, applying It$\hat{o}$’s formula to get

(15) $$\begin{array}{c} dG_{2}=\left[\mu -\left(\alpha +\mu \right)S+F_{2}-\left(\gamma_{1}\left(1-e^{-\mu \tau_{1}}\right)+\mu \right)I\right]dt+\sigma_{1}SdW_{1}+\sigma_{3}IdW_{3}. \end{array}$$

Taking the integral for (15) over [0,t], dividing by *t* and utilizing (6), we have

(16) $$\begin{array}{ccc} \frac{G_{2}\left(t\right)-G_{2}\left(0\right)}{t} & = & \mu -\left(\alpha +\mu \right)\langle S\left(t\right)\rangle +\langle F_{2}\left(t\right)\rangle -\left(\gamma_{1}\left(1-e^{-\mu \tau_{1}}\right)+\mu \right)\langle I\left(t\right)\rangle \\ & + & \frac{\sigma_{1}}{t}\int_{0}^{t}S\left(s\right)dW_{1}+\frac{\sigma_{3}}{t}\int_{0}^{t}I\left(s\right)dW_{3}\\ & \leq & \mu -\left(\alpha +\mu \right)\langle S\left(t\right)\rangle +\frac{\beta_{2}\alpha \mu }{\eta_{1}\alpha \mu +c_{2}}\langle I_{2}\left(t\right)\rangle -\left(\gamma_{1}\left(1-e^{-\mu \tau_{1}}\right)+\mu \right)\langle I\left(t\right)\rangle \\ & + & \frac{\sigma_{1}}{t}\int_{0}^{t}S\left(s\right)dW_{1}+\frac{\sigma_{3}}{t}\int_{0}^{t}I\left(s\right)dW_{3}. \end{array}$$

From (16), we get

(17) $$\begin{array}{c} \langle S\left(t\right)\rangle \leq \frac{\mu }{\alpha +\mu }-\frac{1}{\alpha +\mu }\left(\frac{c_{1}}{\alpha +\mu }-\frac{\beta_{2}\alpha \mu }{\eta_{1}\alpha \mu +c_{2}}\right)\langle I\left(t\right)\rangle +\frac{1}{\alpha +\mu }M_{1}\left(t\right), \end{array}$$

where $c_{1}=\left(\gamma_{1}\left(1-e^{-\mu \tau_{1}}\right)+\mu \right)\left(\alpha +\mu \right)$ and $M_{1}\left(t\right)=\frac{G_{2}\left(0\right)-G_{2}\left(t\right)}{t}+\frac{\sigma_{1}}{t}\int_{0}^{t}S\left(s\right)dW_{1}+$$\frac{\sigma_{3}}{t}\int_{0}^{t}I\left(s\right)dW_{3}.$

Second, we define another Lyapunov function as follows:

$$G_{3}\left(S,V,I\right)=S+V+I+\gamma_{1}e^{-\mu \tau_{1}}\int_{t-\tau_{1}}^{t}I\left(s\right)ds+\gamma_{2}e^{-\mu \tau_{2}}\int_{t-\tau_{2}}^{t}V\left(s\right)ds.$$

Then, by utilizing from It$\hat{a}$ formula, we have

(18) $$\begin{array}{ccc} dG_{3} & = & \left[\mu -\mu S-\left(\gamma_{2}\left(1-e^{-\mu \tau_{2}}\right)+\mu \right)V-\left(\gamma_{1}\left(1-e^{-\mu \tau_{1}}\right)+\mu \right)I\right]dt\\ & + & \sigma_{1}SdW_{1}+\sigma_{2}VdW_{2}+\sigma_{3}IdW_{3}. \end{array}$$

Taking the integral for (18) over [0,t] and dividing by *t*, we get

(19) $$\begin{array}{ccc} \frac{G_{2}\left(t\right)-G_{2}\left(0\right)}{t} & = & \mu -\mu \langle S\left(t\right)\rangle -\left(\gamma_{2}\left(1-e^{-\mu \tau_{2}}\right)+\mu \right)\langle V\left(t\right)\rangle -\left(\gamma_{1}\left(1-e^{-\mu \tau_{1}}\right)+\mu \right)\langle I\left(t\right)\rangle \\ & + & \frac{\sigma_{1}}{t}\int_{0}^{t}S\left(s\right)dW_{1}+\frac{\sigma_{2}}{t}\int_{0}^{t}V\left(s\right)dW_{2}+\frac{\sigma_{3}}{t}\int_{0}^{t}I\left(s\right)dW_{3}. \end{array}$$

From (19), we obtain

(20) $$\begin{array}{c} \langle V\left(t\right)\rangle =\frac{\mu (\alpha +\mu )}{c_{2}}-\frac{\mu (\alpha +\mu )}{c_{2}}\langle S\left(t\right)\rangle -\frac{c_{1}}{c_{2}}\langle I\left(t\right)\rangle +\frac{\mu (\alpha +\mu )}{c_{2}}M_{2}\left(t\right), \end{array}$$

where $M_{2}\left(t\right)=\frac{G_{2}\left(0\right)-G_{2}\left(t\right)}{t}+\frac{\sigma_{1}}{t}\int_{0}^{t}S\left(s\right)dW_{1}+\frac{\sigma_{2}}{t}\int_{0}^{t}V\left(s\right)dW_{2}+\frac{\sigma_{3}}{t}\int_{0}^{t}I\left(s\right)dW_{3}.$

It follows from Lemmas 1 and 2 that $\lim_{t\to \infty }M_{1}\left(t\right)=\lim_{t\to \infty }M_{2}\left(t\right)=0$ a.s.

Third, we define $G_{4}\left(t\right)=\ln I\left(t\right)$, then employing It$\hat{o}$’s formula and using (6), (8) will lead us to the following:

(21) $$\begin{array}{ccc} dG_{4} & = & \left[{\bar{F}}_{1}+{\bar{F}}_{2}-\left(\gamma_{1}+\mu +\frac{\sigma_{3}^{2}}{2}\right)\right]dt+\sigma_{3}dW_{3}\\ & \leq & \left[\frac{\beta_{1}\mu }{(1+\lambda_{1})\mu +\alpha }+\frac{\beta_{2}\alpha \mu }{\eta_{1}\alpha \mu +c_{2}}-\left(\gamma_{1}+\mu +\frac{\sigma_{3}^{2}}{2}\right)\right]dt+\sigma_{3}dW_{3}. \end{array}$$

Integrating the inequality (21) over [0,t] and dividing by *t*, we get

(22) $$\begin{array}{c} \frac{\ln I(t)}{t}\leq \left(\gamma_{1}+\mu +\frac{\sigma_{3}^{2}}{2}\right)\left(R_{0}^{s}-1\right)+\frac{\ln I(0)}{t}+\frac{\sigma_{3}W_{3}\left(t\right)}{t}. \end{array}$$

According to the large number theorem for martingale (see Theorem 3.4 in [26]), we have $\lim_{t\to \infty }\frac{W_{3}\left(t\right)}{t}=0$ a.s. So, if $R_{0}^{s}<1,$ we conclude that

(23) $$\begin{array}{c} \lim_{t\to \infty }\sup\frac{\ln I(t)}{t}\leq \left(\gamma_{1}+\mu +\frac{\sigma_{3}^{2}}{2}\right)\left(R_{0}^{s}-1\right)<0\;\;a.s. \end{array}$$

The obtained result in (23) leads to $\lim_{t\to \infty }I\left(t\right)=0,$ and then $\lim_{t\to \infty }\langle I\left(t\right)\rangle =0$ a.s. Therefore, from (17) and (20), we obtain

$$\lim_{t\to \infty }\langle S\left(t\right)\rangle \leq \frac{\mu }{\alpha +\mu },\;\;\;\;\;\;\;\lim_{t\to \infty }\langle V\left(t\right)\rangle \leq \frac{\alpha \mu }{c_{2}}.$$

□

### 2.3. Persistence of Disease in Mean

**Definition** **1.** *We say that model (3) is persistent in the mean, if*

$$\lim_{t\to \infty }\inf\langle S\left(t\right)\rangle >0,\lim_{t\to \infty }\inf\langle V\left(t\right)\rangle >0,\;\;\mathrm{and}\lim_{t\to \infty }\inf\langle I\left(t\right)\rangle >0\;\;\;a.s.$$

**Lemma** **3.** *(Lemma 5.1 [27]) Let f∈C([0,+∞)×Ω,(0,+∞)). If there exist $\rho_{1},\rho_{2}>0,$ s.t.*

$$\ln f\left(t\right)\geq \rho_{1}t-\rho_{2}\int_{0}^{t}f\left(s\right)ds+F\left(t\right),\forall t\geq 0,$$

*where F∈C([0,+∞)×Ω,R) and $\lim_{t\to \infty }\frac{F(t)}{t}=0$ a.s., then*

$$\lim_{t\to \infty }\inf\langle f\left(t\right)\rangle \geq \frac{\rho_{1}}{\rho_{2}}\;\;\;\;\;\;a.s.$$

In the forthcoming theorem, we determine the sufficient conditions for the persistence of disease for the model (3) based on Lemma 3.

**Theorem** **3.** *Let (S(t),V(t),I(t)) be the solution of model (3) with initial value $S\left(0\right)>0,V\left(\zeta_{2}\right)$≥0 and $I(\zeta_{1})\geq 0$, $\forall \zeta_{1}\in \left[-\tau_{1},0\right)$ and $\zeta_{2}\in \left[-\tau_{2},0\right)$ with V(0)>0 and I(0)>0. Under the condition*

(24) $$\frac{c_{1}}{\alpha +\mu }-\frac{\beta_{2}\alpha \mu }{\eta_{1}\alpha \mu +c_{2}}>0,$$

*if $R_{0}^{s}>1,$ then the solution is said to be persistent in the mean and the following properties are satisfied:*

- $\lim_{t\to \infty }\inf\langle I\left(t\right)\rangle \geq \frac{\left(\gamma_{1}+\mu +\frac{\sigma_{3}^{2}}{2}\right)\left(R_{0}^{s}-1\right)}{L_{1}+L_{2}}=I^{*},$ where

$L_{1}=\frac{\beta_{1}\mu }{(1+\lambda_{1})\mu +\alpha }\left(\lambda_{2}+\lambda_{3}\frac{\mu }{\alpha +\mu }\right)+\frac{\beta_{2}\alpha \mu }{\eta_{1}\alpha \mu +c_{2}}\left(\eta_{2}+\eta_{3}\frac{\alpha \mu }{c_{2}}\right),\;\;L_{2}=\frac{\beta_{2}\alpha \left(\beta_{2}\alpha \mu \left(\alpha +\mu \right)+c_{1}\left(\eta_{1}\alpha \mu +c_{2}\right)\right)}{{\left(\eta_{1}\alpha \mu +c_{2}\right)}^{2}\left(\alpha +\mu \right)};$

-
$\lim_{t\to \infty }\sup\langle S\left(t\right)\rangle \leq \frac{\mu }{\alpha +\mu }-\left(\frac{c_{1}}{\alpha +\mu }-\frac{\beta_{2}\alpha \mu }{\eta_{1}\alpha \mu +c_{2}}\right)I^{*}=S^{*};$

-
$\lim_{t\to \infty }\sup\langle V\left(t\right)\rangle =\frac{\mu (\alpha +\mu )}{c_{2}}\left(1-S^{*}\right)-\frac{c_{1}}{c_{2}}I^{*}=V^{*}.$

**Proof.**  Applying It$\hat{o}$’s formula again on $G_{4}\left(t\right)$ and using (7), (9), we have

(25) $$\begin{array}{ccc} dG_{4} & = & \left[{\bar{F}}_{1}+{\bar{F}}_{2}-\left(\gamma_{1}+\mu +\frac{\sigma_{3}^{2}}{2}\right)\right]dt+\sigma_{3}dW_{3}\\ & \geq & [\frac{\beta_{1}\mu }{(1+\lambda_{1})\mu +\alpha }+\frac{\beta_{2}c_{2}V}{\eta_{1}\alpha \mu +c_{2}}-\left(\gamma_{1}+\mu +\frac{\sigma_{3}^{2}}{2}\right)\\ & - & \left(\frac{\beta_{1}\mu }{(1+\lambda_{1})\mu +\alpha }\left(\lambda_{2}+\lambda_{3}\frac{\mu }{\alpha +\mu }\right)+\frac{\beta_{2}\alpha \mu }{\eta_{1}\alpha \mu +c_{2}}\left(\eta_{2}+\eta_{3}\frac{\alpha \mu }{c_{2}}\right)\right)I]dt+\sigma_{3}dW_{3}. \end{array}$$

Integrating inequality (25) over [0,t], and dividing by *t*, one can obtain

(26) $$\begin{array}{ccc} \frac{G_{4}\left(t\right)-G_{4}\left(0\right)}{t} & \geq & \frac{\beta_{1}\mu }{(1+\lambda_{1})\mu +\alpha }+\frac{\beta_{2}c_{2}}{\eta_{1}\alpha \mu +c_{2}}\langle V\left(t\right)\rangle -\left(\gamma_{1}+\mu +\frac{\sigma_{3}^{2}}{2}\right)-L_{1}\langle I\left(t\right)\rangle +\frac{\sigma_{3}W_{3}\left(t\right)}{t}\\ & = & \frac{\beta_{1}\mu }{(1+\lambda_{1})\mu +\alpha }-\left(\gamma_{1}+\mu +\frac{\sigma_{3}^{2}}{2}\right)+\frac{\beta_{2}\left(\alpha +\mu \right)\mu }{\eta_{1}\alpha \mu +c_{2}}\\ & - & \frac{\beta_{2}\left(\alpha +\mu \right)\mu }{\eta_{1}\alpha \mu +c_{2}}\langle S\left(t\right)\rangle -\frac{\beta_{2}c_{1}}{\eta_{1}\alpha \mu +c_{2}}\langle I\left(t\right)\rangle +\frac{\beta_{2}\left(\alpha +\mu \right)}{\eta_{1}\alpha \mu +c_{2}}M_{2}-L_{1}\langle I\left(t\right)\rangle +\frac{\sigma_{3}W_{3}}{t}\\ & \geq & \frac{\beta_{1}\mu }{(1+\lambda_{1})\mu +\alpha }+\frac{\beta_{2}\alpha \mu }{\eta_{1}\alpha \mu +c_{2}}-\left(\gamma_{1}+\mu +\frac{\sigma_{3}^{2}}{2}\right)\\ & - & \left[\frac{\beta_{2}c_{1}}{\eta_{1}\alpha \mu +c_{2}}-\frac{\beta_{2}\mu }{\eta_{1}\alpha \mu +c_{2}}\left(\frac{c_{1}}{\alpha +\mu }-\frac{\beta_{2}\alpha \mu }{\eta_{1}\alpha \mu +c_{2}}\right)\right]\langle I\left(t\right)\rangle \\ & - & L_{1}\langle I\left(t\right)\rangle +\frac{\beta_{2}\mu }{\eta_{1}\alpha \mu +c_{2}}M_{1}+\frac{\beta_{2}\left(\alpha +\mu \right)}{\eta_{1}\alpha \mu +c_{2}}M_{2}+\frac{\sigma_{3}W_{3}}{t}\\ & = & \frac{\beta_{1}\mu }{(1+\lambda_{1})\mu +\alpha }+\frac{\beta_{2}\alpha \mu }{\eta_{1}\alpha \mu +c_{2}}-\left(\gamma_{1}+\mu +\frac{\sigma_{3}^{2}}{2}\right)\\ & - & \frac{\beta_{2}\alpha \left[\beta_{2}\alpha \mu \left(\alpha +\mu \right)+c_{1}\left(\eta_{1}\alpha \mu +c_{2}\right)\right]}{{\left(\eta_{1}\alpha \mu +c_{2}\right)}^{2}\left(\alpha +\mu \right)}\langle I\left(t\right)\rangle \\ & - & L_{1}\langle I\left(t\right)\rangle +\frac{\beta_{2}\mu }{\eta_{1}\alpha \mu +c_{2}}M_{1}+\frac{\beta_{2}\left(\alpha +\mu \right)}{\eta_{1}\alpha \mu +c_{2}}M_{2}+\frac{\sigma_{3}W_{3}}{t}. \end{array}$$

From (26), we get

(27) $$\begin{array}{c} \ln I\left(t\right)\geq \left(\gamma_{1}+\mu +\frac{\sigma_{3}^{2}}{2}\right)\left(R_{0}^{s}-1\right)t-\left(L_{1}+L_{2}\right)\langle I\left(t\right)\rangle t+M\left(t\right), \end{array}$$

where

$$M\left(t\right)=\frac{\beta_{2}\mu \;t}{\eta_{1}\alpha \mu +c_{2}}M_{1}+\frac{\beta_{2}\left(\alpha +\mu \right)\;t}{\eta_{1}\alpha \mu +c_{2}}M_{2}+\sigma_{3}W_{3}+\ln I\left(0\right).$$

Since, $\lim_{t\to \infty }M_{1}\left(t\right)=\lim_{t\to \infty }M_{2}\left(t\right)=\lim_{t\to \infty }\frac{\sigma_{3}W_{3}}{t}=0,$ hence $\lim_{t\to \infty }\frac{M(t)}{t}=0$ a.s. According to Lemma 3 and inequality (27), we conclude

$$\lim_{t\to \infty }\inf\langle I\left(t\right)\rangle \geq \frac{\left(\gamma_{1}+\mu +\frac{\sigma_{3}^{2}}{2}\right)\left(R_{0}^{s}-1\right)}{L_{1}+L_{2}}=I^{*}.$$

Now, it follows from (16) that

(28) $$\begin{array}{ccc} \lim_{t\to \infty }\sup\langle S\left(t\right)\rangle & \leq & \frac{\mu }{\alpha +\mu }-\frac{1}{\alpha +\mu }\left(\frac{c_{1}}{\alpha +\mu }-\frac{\beta_{2}\alpha \mu }{\eta_{1}\alpha \mu +c_{2}}\right)\lim_{t\to \infty }\inf\langle I\left(t\right)\rangle -\frac{1}{\alpha +\mu }\lim_{t\to \infty }M_{1}\left(t\right)\\ & = & \frac{\mu }{\alpha +\mu }-\frac{1}{\alpha +\mu }\left(\frac{c_{1}}{\alpha +\mu }-\frac{\beta_{2}\alpha \mu }{\eta_{1}\alpha \mu +c_{2}}\right)I^{*}=S^{*}. \end{array}$$

Also, from (20), we get

(29) $$\begin{array}{ccc} \lim_{t\to \infty }\sup\langle V\left(t\right)\rangle & = & \frac{\mu (\alpha +\mu )}{c_{2}}\left(1-\lim_{t\to \infty }\sup\langle S\left(t\right)\rangle -\lim_{t\to \infty }M_{2}\left(t\right)\right)-\lim_{t\to \infty }\sup\frac{c_{1}}{c_{2}}\langle I\left(t\right)\rangle \\ & = & \frac{\mu (\alpha +\mu )}{c_{2}}\left(1-S^{*}\right)-\frac{c_{1}}{c_{2}}I^{*}=V^{*}. \end{array}$$

The proof is completed. □

## 3. Numerical Model Analysis

In this section, by constructing two effective methods that give dynamically consistent solutions with the continuous-time model, we intend to demonstrate the validity and effectiveness of the studied results. The first method is SSTM, and we are considering it here because it is computationally simplified. The second method will be modern, designed by incorporating the split-step θ method with a nonstandard finite difference method for the model (3) and called the SSSTNSFD method. Our modern method is constructed based on Mickens’framework, where it is free of any numerical instabilities regardless of the size of the step-size used in the numerical simulation. In what follows, $0\leq \theta \leq 1,m_{1}=\frac{\tau_{1}}{h},m_{2}=\frac{\tau_{2}}{h},$ where for $\bar{N}\in N,h=\frac{T}{\bar{N}}$ represents the time step size on [0,T], and $n=0,1,2,...,\bar{N}.$ Moreover, for i∈{1,2,3},$\Delta W_{i,n}=\sqrt{h}\;\;\xi_{i,n}$ where $\xi_{i,n}$ is independent Gaussian random variable N(0,1).

### 3.1. Split-Step θ-Milstein Scheme

The proposed SSTM method is easy to construct to get an approximate solution for the model (3), which was designed and used for the first time in [28]. Therefore, it is directly constructed from the model (3) as follows:

(30) $$\left\{\begin{array}{cc} S^{n+1} & =S^{n}+(\mu -\left(\mu +\alpha \right)S^{n}+\gamma_{1}e^{-\mu \tau_{1}}\left(\theta I^{n-m_{1}+1}+\left(1-\theta \right)I^{n-m_{1}}\right)\\ \; & -\frac{\beta_{1}S^{n}I^{n}}{1+\lambda_{1}S^{n}+\lambda_{2}I^{n}+\lambda_{3}S^{n}I^{n}})h+\sigma_{1}S^{n}\Delta W_{1,n}+\frac{\sigma_{1}^{2}}{2}S^{n}\left(\Delta W_{1,n}^{2}-h\right),\\ V^{n+1} & =V^{n}+(\alpha S^{n+1}-\left(\mu +\gamma_{2}\right)V^{n}+\gamma_{2}e^{-\mu \tau_{2}}\left(\theta V^{n-m_{2}+1}+\left(1-\theta \right)V^{n-m_{2}}\right)\\ \; & -\frac{\beta_{2}V^{n}I^{n}}{1+\eta_{1}V^{n}+\eta_{2}I^{n}+\eta_{3}V^{n}I^{n}})h+\sigma_{2}V^{n}\Delta W_{2,n}+\frac{\sigma_{2}^{2}}{2}V^{n}\left(\Delta W_{2,n}^{2}-h\right),\\ I^{n+1} & =I^{n}+(\frac{\beta_{1}S^{n+1}I^{n}}{1+\lambda_{1}S^{n+1}+\lambda_{2}I^{n}+\lambda_{3}S^{n+1}I^{n}}+\frac{\beta_{2}V^{n+1}I^{n}}{1+\eta_{1}V^{n+1}+\eta_{2}I^{n}+\eta_{3}V^{n+1}I^{n}}\\ \; & -\left(\mu +\gamma_{1}\right)I^{n})h+\sigma_{3}I^{n}\Delta W_{3,n}+\frac{\sigma_{3}^{2}}{2}I^{n}\left(\Delta W_{3,n}^{2}-h\right). \end{array}\right.$$

### 3.2. Stochastic Split-Step θ-Nonstandard Finite Difference Method

Specifically, this section aims to design and analyze a dynamically consistent SSSTNSFD method to obtain an approximate solution of the model (3), where this newly constructed method enjoys the properties of elementary stability and preservation of the positivity of solution regardless of the size of “h” used in the numerical simulations. Therefore, in order to construct our new scheme in the sense of Mickens (see [29,30]), we use non-local approximations (i.e., the terms on the right hand side of model (3) must be contain terms with the form $S^{n+1},S^{n},V^{n+1},V^{n},I^{n+1}$ and $I^{n}$), and the terms which contain time delay are approximated by split-step θ method (i.e., the term $I(t-\tau_{1})$ is approximated by $\theta I^{n-m_{1}+1}+\left(1-\theta I^{n-m_{1}}\right),$ and $V(t-\tau_{2})$ is approximated by $\theta V^{n-m_{2}+1}+\left(1-\theta V^{n-m_{2}}\right)$). Moreover, for any $g\left(t\right)\in C^{1}\left(R\right),$ we choose an equivalent derivative which can be defined as $\frac{dg}{dt}=\frac{g^{n+1}-g^{n}}{v(h)},$ where v(h) is a real-valued nonnegative function called the denominator function in which satisfies the condition $v\left(h\right)=h+O\left(h^{2}\right).$ Therefore, for any h>0, a common function such $v\left(h\right)=1-e^{-h}$ can be used. Consequently, the SSSTNSFD scheme is constructed as:

(31) $$\begin{array}{ccc} S^{n+1}-S^{n} & = & v\left(h\right)[\mu -\left(\mu +\alpha \right)S^{n+1}+\gamma_{1}e^{-\mu \tau_{1}}\left(\theta I^{n-m_{1}+1}+\left(1-\theta \right)I^{n-m_{1}}\right)\\ & - & \frac{\beta_{1}S^{n+1}I^{n}}{1+\lambda_{1}S^{n}+\lambda_{2}I^{n}+\lambda_{3}S^{n}I^{n}}]+\sigma_{1}S^{n}\Delta W_{1,n},\\ V^{n+1}-V^{n} & = & v\left(h\right)[\alpha S^{n+1}-\left(\mu +\gamma_{2}\right)V^{n+1}+\gamma_{2}e^{-\mu \tau_{2}}\left(\theta V^{n-m_{2}+1}+\left(1-\theta \right)V^{n-m_{2}}\right)\\ & - & \frac{\beta_{2}V^{n+1}I^{n}}{1+\eta_{1}V^{n}+\eta_{2}I^{n}+\eta_{3}V^{n}I^{n}}]+\sigma_{2}V^{n}\Delta W_{2,n},\\ I^{n+1}-I^{n} & = & v\left(h\right)[\frac{\beta_{1}S^{n+1}I^{n}}{1+\lambda_{1}S^{n+1}+\lambda_{2}I^{n}+\lambda_{3}S^{n+1}I^{n}}+\frac{\beta_{2}V^{n+1}I^{n}}{1+\eta_{1}V^{n+1}+\eta_{2}I^{n}+\eta_{3}V^{n+1}I^{n}}\\ & - & \left(\mu +\gamma_{1}\right)I^{n+1}]+\sigma_{3}I^{n}\Delta W_{3,n}. \end{array}$$

Thence, re-arranging equations in (31) yields the following explicit SSSTNSFD scheme for model (3):

(32) $$\left\{\begin{array}{cc} S^{n+1} & =\frac{S^{n}+\left[\mu +\gamma_{1}e^{-\mu \tau_{1}}\left(\theta I^{n-m_{1}+1}+\left(1-\theta \right)I^{n-m_{1}}\right)\right]v\left(h\right)+\sigma_{1}S^{n}\Delta W_{1,n}}{1+\left[\alpha +\mu +\frac{\beta_{1}I^{n}}{1+\lambda_{1}S^{n}+\lambda_{2}I^{n}+\lambda_{3}S^{n}I^{n}}\right]v\left(h\right)},\\ V^{n+1} & =\frac{V^{n}+\left[\alpha S^{n+1}+\gamma_{2}e^{-\mu \tau_{2}}\left(\theta V^{n-m_{2}+1}+\left(1-\theta \right)V^{n-m_{2}}\right)\right]v\left(h\right)+\sigma_{2}V^{n}\Delta W_{2,n}}{1+\left[\gamma_{2}+\mu +\frac{\beta_{2}I^{n}}{1+\eta_{1}V^{n}+\eta_{2}I^{n}+\eta_{3}V^{n}I^{n}}\right]v\left(h\right)},\\ I^{n+1} & =\frac{I^{n}\left(1+\left[\frac{\beta_{1}S^{n+1}}{1+\lambda_{1}S^{n+1}+\lambda_{2}I^{n}+\lambda_{3}S^{n+1}I^{n}}+\frac{\beta_{2}V^{n+1}}{1+\eta_{1}V^{n+1}+\eta_{2}I^{n}+\eta_{3}V^{n+1}I^{n}}\right]v\left(h\right)+\sigma_{3}\Delta W_{3,n}\right)}{1+\left(\gamma_{1}+\mu \right)v\left(h\right)}, \end{array}\right.$$

It should be noted that one of the essential features of the SSSTNSFD method (32) is represented in its ease of implementation since the numerical computation of the discrete solutions of the model (3) is carried out explicitly by the following sequential process, which is mainly similar to Gauss–Seidel-method, where, we first compute $S^{n+1},$ then $V^{n+1},$ and then $I^{n+1}.$ It is worth mentioning that, in the implementation of the above method (32), once a variable (e.g., $S^{n+1}$ ) is computed, it is instantly used for the computations of subsequent variables (e.g., $S^{n+1}$ is used for the computation of $V^{n+1},$ and then, both are used for the computation of $I^{n+1}).$ In fact, this asserts the Gauss–Seidel natural of implementing the SSSTNSFD method.

#### Convergence Analysis of Split-Step θ-Nonstandard Finite Difference Method

**Theorem** **4.** *With $n,m_{1},m_{2}\geq 0,$ and $\tau_{1},\tau_{2}>0,$ the scheme (32) admits a unique nonnegative solution $\left(S^{n},V^{n},I^{n}\right)\in R_{+}^{3}$ for any initial value $S\left(0\right)>0,V\left(\zeta_{2}\right)\geq 0,\forall \zeta_{2}\in \left[-\tau_{2},0\right)$ and $I\left(\zeta_{1}\right)\geq 0,\forall \zeta_{1}\in \left[-\tau_{1},0\right)$ with V(0)>0 and I(0)>0.*

**Proof.**  The proof of the current theorem can be obtained straightforwardly due to the fact that the constraint of biological problems is always positive. □

**Theorem** **5.** Let the initial data of (32) hold the inequality $S^{0}+V^{0}+I^{0}\leq T^{*},$ where $T^{*}=\frac{\mu (\gamma_{2}\left(1-e^{-\mu \tau_{2}}\right)+\mu )+\alpha \mu }{c_{2}}.$ Then, for $n=0,1,2,...,\bar{N},$ there exists a constant

$$M_{n}=\left(1+\sum_{i=1}^{n}\prod_{j=1}^{i}\left(1+\sigma \Delta W_{n+1-j}\right)\right)\mu v\left(h\right)+\prod_{j=0}^{n}\left(1+\sigma \Delta W_{j}\right)T^{*}>0,$$

*in which $S^{n+1},V^{n+1},I^{n+1}\leq M_{n}.$*

**Proof.**  We start our proof by rewriting equations in (31) as follows:

(33) $$\begin{array}{ccc} S^{n+1}-S^{n} & = & v\left(h\right)[\mu -\left(\mu +\alpha \right)S^{n}+\gamma_{1}e^{-\mu \tau_{1}}\left(\theta I^{n-m_{1}+1}+\left(1-\theta \right)I^{n-m_{1}}\right)\\ & - & \frac{\beta_{1}S^{n}I^{n}}{1+\lambda_{1}S^{n}+\lambda_{2}I^{n}+\lambda_{3}S^{n}I^{n}}]+\sigma_{1}S^{n}\Delta W_{1,n},\\ V^{n+1}-V^{n} & = & v\left(h\right)[\alpha S^{n}-\left(\mu +\gamma_{2}\right)V^{n}+\gamma_{2}e^{-\mu \tau_{2}}\left(\theta V^{n-m_{2}+1}+\left(1-\theta \right)V^{n-m_{2}}\right)\\ & - & \frac{\beta_{2}V^{n}I^{n}}{1+\eta_{1}V^{n}+\eta_{2}I^{n}+\eta_{3}V^{n}I^{n}}]+\sigma_{2}V^{n}\Delta W_{2,n},\\ I^{n+1}-I^{n} & = & v\left(h\right)[\frac{\beta_{1}S^{n}I^{n}}{1+\lambda_{1}S^{n}+\lambda_{2}I^{n}+\lambda_{3}S^{n}I^{n}}+\frac{\beta_{2}V^{n}I^{n}}{1+\eta_{1}V^{n}+\eta_{2}I^{n}+\eta_{3}V^{n}I^{n}}\\ & - & \left(\mu +\gamma_{1}\right)I^{n}]+\sigma_{3}I^{n}\Delta W_{3,n}. \end{array}$$

By adding the above equations, we get

(34) $$\begin{array}{ccc} S^{n+1}+V^{n+1}+I^{n+1} & = & S^{n}+V^{n}+I^{n}+v\left(h\right)[\mu -\mu \left(S^{n}+V^{n}+I^{n}\right)-(\gamma_{1}I^{n}+\gamma_{2}V^{n}\\ & - & \gamma_{1}e^{-\mu \tau_{1}}\left(\theta I^{n-m_{1}+1}+\left(1-\theta \right)I^{n-m_{1}}\right)-\gamma_{2}e^{-\mu \tau_{2}}(\theta V^{n-m_{2}+1}\\ & + & \left(1-\theta \right)V^{n-m_{2}}))]+\sigma_{1}S^{n}\Delta W_{1,n}+\sigma_{2}V^{n}\Delta W_{2,n}+\sigma_{3}I^{n}\Delta W_{3,n}. \end{array}$$

Suppose that $\sigma =\max\{\sigma_{1},\sigma_{2},\sigma_{3}\}$ and $\Delta W_{n}=\max\left\{\Delta W_{1,n},\Delta W_{2,n},\Delta W_{3,n}\right\}.$ Thus, from (34) we get

(35) $$\begin{array}{ccc} S^{n+1}+V^{n+1}+I^{n+1} & \leq & \mu v\left(h\right)+\left(1+\sigma \Delta W_{n}\right)\left(S^{n}+V^{n}+I^{n}\right). \end{array}$$

Now, using inequality (35), it follows that for n=0, we get

$$\begin{array}{ccc} S^{1}+V^{1}+I^{1} & \leq & \mu v\left(h\right)+\left(1+\sigma \Delta W_{0}\right)T^{*}=M_{0}>T^{*}. \end{array}$$

Next, for n=1, we obtain

$$\begin{array}{ccc} S^{2}+V^{2}+I^{2} & \leq & \mu v\left(h\right)+\left(1+\sigma \Delta W_{1}\right)M_{0}\\ & = & \left(1+\left(1+\sigma \Delta W_{1}\right)\right)\mu v\left(h\right)+\left(1+\sigma \Delta W_{1}\right)\left(1+\sigma \Delta W_{0}\right)T^{*}=M_{1}>M_{0}. \end{array}$$

Also, for n=2, we get

$$\begin{array}{ccc} S^{3}+V^{3}+I^{3} & \leq & \mu v\left(h\right)+\left(1+\sigma \Delta W_{2}\right)M_{1}\\ & = & \left(1+\left(1+\sigma \Delta W_{2}\right)+\left(1+\sigma \Delta W_{2}\right)\left(1+\sigma \Delta W_{1}\right)\right)\mu v\left(h\right)\\ & + & \left(1+\sigma \Delta W_{2}\right)\left(1+\sigma \Delta W_{1}\right)\left(1+\sigma \Delta W_{0}\right)T^{*}=M_{2}>M_{1}. \end{array}$$

Therefore, for $0\leq n\leq \bar{N},$ where $M_{-1}=T^{*},$ we deduce

$$\begin{array}{ccc} S^{n+1}+V^{n+1}+I^{n+1} & \leq & \mu v\left(h\right)+\left[1+\sigma \Delta W_{n}\right]M_{n-1}=M_{n}>M_{n-1}, \end{array}$$

and this in turn proves that $S^{n+1},V^{n+1},I^{n+1}\leq M_{n},\forall n\in \left\{0,1,2,...,\bar{N}\right\}.$ □

**Definition** **2.** *The SSSTNSFD scheme (32) is said to be asymptotically stable, if there exist positive constants $C_{i},i\in N_{1}^{3},$ s.t., with any initial value $S\left(0\right)>0,V\left(\zeta_{2}\right)\geq 0,\forall \zeta_{2}\in \left[-\tau_{2},0\right)$ and $I\left(\zeta_{1}\right)\geq 0,\forall \zeta_{1}\in \left[-\tau_{1},0\right)$ with V(0)>0 and I(0)>0, the following hold: $S^{n+1}\leq C_{1},\;\;\;V^{n+1}\leq C_{2},\;\;\;I^{n+1}\leq C_{3},\forall n\geq 0.$*

**Theorem** **6.** *With the same hypothesis of Theorems 4 and 5, the modern SSSTNSFD scheme (32) is asymptotically stable.*

**Proof.**  According to Theorem 4, the numerical solution of (32) is nonnegative, and based on Theorem 5, the solution is bounded. Therefore, for all n≥0, we can find positive constants such $C_{i},i\in N_{1}^{3},$ s.t. $S^{n+1}\leq C_{1},\;\;\;V_{1}^{n+1}\leq C_{2},\;\;\;I_{1}^{n+1}\leq C_{3}.$ The proof is completed. □

### 3.3. Illustration and Discussion

In this section, after constructing the explicit SSTM and SSSTNSFD schemes for (3), we have two aims. The first aim is to use these schemes to show the validity of the results obtained in Section 2, as well as to discuss the effects of some parameters on the dynamics of the model (3). The second aim is to compare the two schemes in terms of dynamic properties, such as positivity, boundedness, and consistency, to show the new method’s efficiency, especially when applied to larger time step.

We consider the initial values S(0)=0.3,V(0)=0.3,I(0)=0.2, besides the parameter values in the set (36) to achieve our goals.

(36) $$\begin{array}{cc} & \{\mu =1,\alpha =10,\gamma_{1}=1.5,\gamma_{2}=2.4,\theta =0.5,\tau_{1}=1,\tau_{2}=2,\sigma_{1}=0.1,\sigma_{2}=0.02,\\ & \sigma_{3}=0.05,\lambda_{1}=0.2,\lambda_{2}=0.3,\lambda_{3}=0.4,\eta_{1}=0.4,\eta_{2}=0.2,\eta_{3}=0.1\}. \end{array}$$

**Figure 1:**
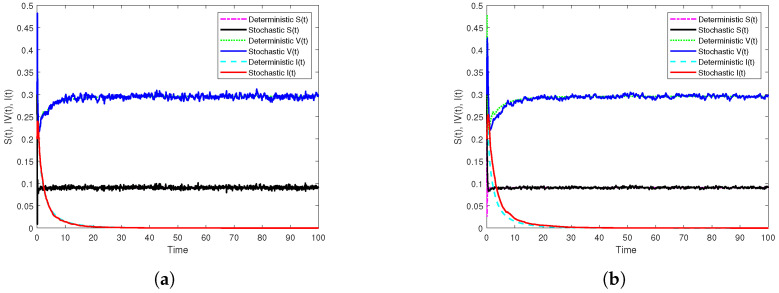
Numerical simulations of the extinction results of model (3) by the two constructed methods with h=0.1 on $\left[0,10^{2}\right]$ compared with the corresponding deterministic model. (**a**) The simulation of S,V and I paths by SSTM method. (**b**) The simulation of S,V and I paths by SSSTNSFD method.

**Figure 2:**
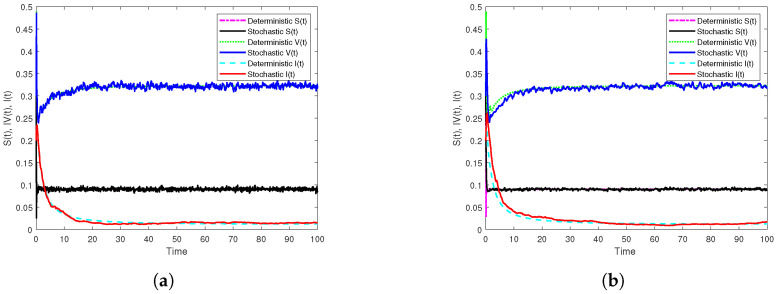
Numerical simulations illustrate the effect of $\tau_{1},\tau_{2}$ on the dynamics of model (3) by the two constructed methods with h=0.1 on $\left[0,10^{2}\right]$ compared with the corresponding deterministic model. (**a**) The simulation of S,V and I paths by SSTM method. (**b**) The simulation of S,V and I paths by SSSTNSFD method.

**Figure 3:**
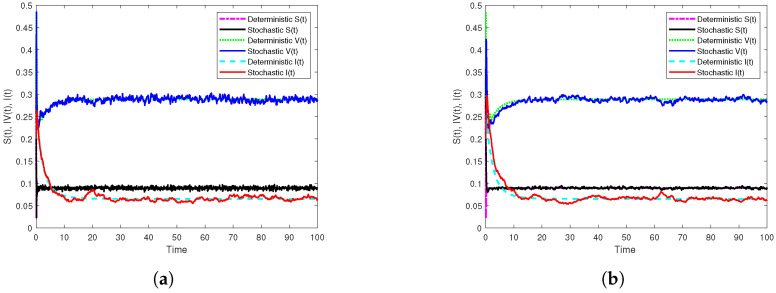
Numerical simulations illustrate the effect of parameters of incidence rates on the dynamics of model (3) by the two constructed methods with h=0.1 on $\left[0,10^{2}\right]$ compared with the corresponding deterministic model. (**a**) The simulation of S,V and I paths by SSTM method. (**b**) The simulation of S,V and I paths by SSSTNSFD method.

First, if $\beta_{1}=10.5,\beta_{2}=5.5,$ we get $R_{0}^{s}=0.9561<1.$ In addition, Figure 1a displays the results of Theorem 2 through the generated simulation by SSTM method (30), whilst Figure 1b displays the results of the same theorem through the generated simulation by SSSTNSFD method (32). It is noted that, in both simulations, the disease extincts as long as $R_{0}^{s}<1.$ Moreover, to show the effect of temporary immunities on dynamics of model (3), we only change the length of immunity periods as: $\tau_{1}=0.8,\tau_{2}=1.3,$ and other parameter values kept unaltered. In this case, we find that $R_{0}^{s}=1.0177>1,$ and this indicates that the disease will persist if the temporary immunities are sufficiently small as simulated in Figure 2. Further, in order to show the vital role of the parameters of incidence functions on the dynamics of model (3), we only change the values of the parameters of the incidence functions as: $\lambda_{1}=0.02,\lambda_{2}=0.03,\lambda_{3}=0.04,\eta_{1}=0.04,\eta_{2}=0.02,\eta_{3}=0.01,$ and keeping other parameters fixed, where we get $R_{0}^{s}=1.0994>1.$ It follows from this that disease persists when the parameter values of incidence functions are sufficiently small. The simulations in Figure 3 clarify that through using the two proposed methods.

**Figure 4:**
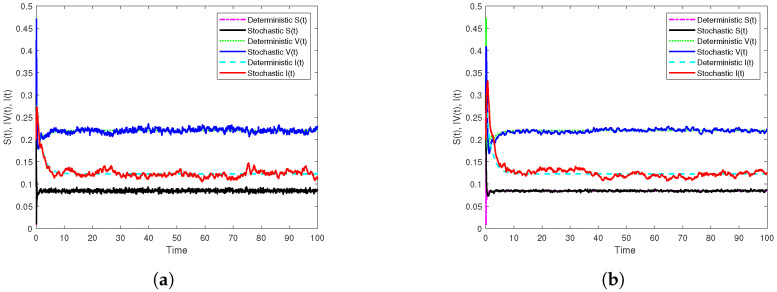
Numerical simulations of the persistence results of model (3) by the two constructed methods with h=0.1 on $\left[0,10^{2}\right]$ compared with the corresponding deterministic model. (**a**) The simulation of S,V and I paths by SSTM method. (**b**) The simulation of S,V and I paths by SSSTNSFD method.

**Figure 5:**
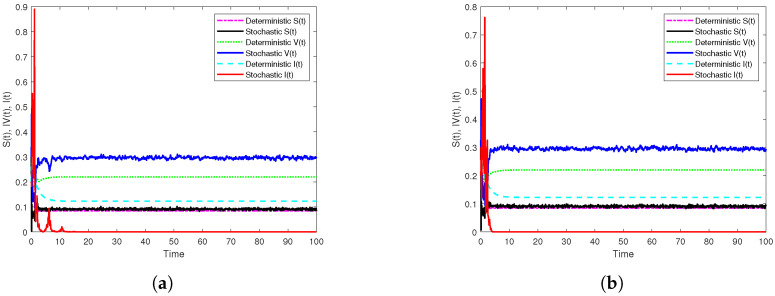
Numerical simulations illustrate the effect of large noise on the dynamics of model (3) by the two methods with $\sigma_{3}=1.5$ and h=0.1 on $\left[0,10^{2}\right]$ compared with the corresponding deterministic model. (**a**) The simulation of S,V and I paths by SSTM method. (**b**) The simulation of S,V and I paths by SSSTNSFD method.

Second, if $\beta_{1}=14,\beta_{2}=7,$ then the reproduction number $R_{0}^{s}=1.2396>1,$ and the condition $\frac{c_{1}}{\alpha +\mu }-\frac{\beta_{2}\alpha \mu }{\eta_{1}\alpha \mu +c_{2}}=0.0977>0$ holds. Therefore, it follows that $I^{*}=0.125,S^{*}=0.0898,$ and $V^{*}=0.2168.$ In addition, Figure 4a displays the results of Theorem 3 through the generated simulation by SSTM method (30), whilst Figure 4b displays the results of the same theorem through the generated simulation by SSSTNSFD method (32). It is noted that, in both simulations, the disease persists as long as $R_{0}^{s}>1.$ Moreover, in order to examine the effectiveness of noise on the dynamics of the model (3), we choose $\sigma_{3}$ to be sufficiently large, e.g., $\sigma_{3}=1.5$ with keeping other parameters unchanged. In this case, we get $R_{0}^{s}=0.8553<1>R_{0}^{d}=1.2402,$ and this reveals that the stochastic model (3) has an extinct disease, while the corresponding deterministic model has an endemic with probability one. This emphasizes that noise can suppress the disease outbreak. The simulations in Figure 5 display this fact.

**Figure 6:**
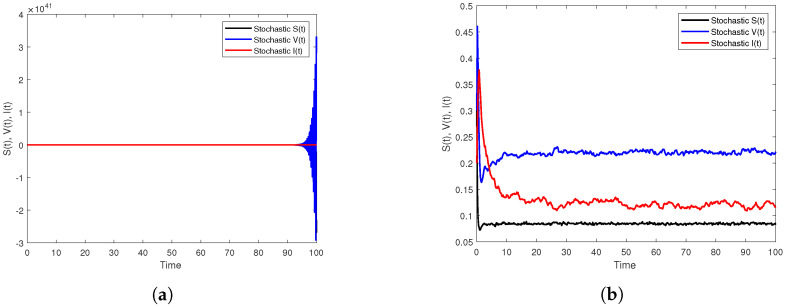
Numerical simulations to compare between the two methods when the size of *h* is increased, e.g., h=0.5 on $\left[0,10^{2}\right]$. (**a**) The simulation of S,V and I paths by SSTM method. (**b**) The simulation of S,V and I paths by SSSTNSFD method.

Finally, in order to compare the two schemes, we increase the size of *h*, e.g., h=0.5, and considering $\beta_{1}=14,\beta_{2}=7,$ with keeping other parameters as mentioned in the set (36). As a result, we observe Figure 6a shows that the SSTM method is extremely sensitive to the time step size and fails to preserve the properties of elementary stability and preservation of positivity of the solution of the model (3) unlike the SSSTNSFD method that adheres to those properties regardless of the size of *h* as demonstrated in Figure 6b. Hence, this reveals the axial role that the time step size plays in detecting the most efficient numerical method.

## 4. Conclusions

Without a doubt, the length of the immunity period plays a significant role in the extinction or persistence of disease, where the short temporary immunity helps in the persistence of the disease, whilst the long-life immunity helps in the extinction of the disease. To support this fact, a new SVIR model has been developed and studied theoretically and numerically. In the theoretical aspect, Lyapunov functions were utilized to prove the existence and uniqueness of the global solution and to establish sufficient conditions for the extinction and persistence of disease. Additionally, the stochastic reproduction number $R_{0}^{s}$ was established, and then we proved that if $R_{0}^{s}$ is less than unity, the disease will die out; if $R_{0}^{s}$ is greater than unity, it will persist in the mean.

In the numerical aspect, a simulation and discussion were conducted by employing both of the two constructed methods to assess the validity of the theoretical results. However, the generated simulations in Figure 2, Figure 3 and Figure 5 have shown the effect of the length of immunity periods, parameters of the incidence rates, and noise on the dynamics of the model, respectively. Based on the comparison between the two used methods, we have observed through Figure 6a that the SSTM method exhibited unexpected results regarding positivity and boundedness of solutions. It was noticed that this method converges only for a small time step size, but when we increased the time step size, the method failed to restore the desired properties. On the other hand, Figure 6b showed that the SSSTNSFD method preserves all the desired properties of the model regardless of the size of the time step size used in the numerical simulation. This supports the idea that the SSSTNSFD scheme is dynamically consistent and more proper for studying the asymptotic dynamics of our model.

## Figures and Tables

**Table 1:** The Biological meanings of parameters of SVIR model.

Parameter	Biological Meaning
μ	The recruitment rate and natural rate of population
α	The rate at which susceptible individuals are moved into the vaccination process
$\beta_{1}$	The transmission coefficient between the two compartments S and I
$\beta_{2}$	The disease transmission rate when the vaccinees contact with infected individuals before obtaining the immunity against the disease
$\gamma_{1}$	The recovery rate of infected individuals
$\gamma_{2}$	The average rate for the vaccinees to obtain immunity and move into recovery compartment
$\tau_{1}$	The length of the immunity period of the recovered infected individuals
$\tau_{2}$	The length of the immunity period of recovered vaccinated individuals

## Data Availability

Not applicable.
